# Machine learning to predict occult metastatic lymph nodes along the recurrent laryngeal nerves in thoracic esophageal squamous cell carcinoma

**DOI:** 10.1186/s12885-023-10670-3

**Published:** 2023-03-02

**Authors:** Yiliang Zhang, Longfu Zhang, Bin Li, Ting Ye, Yang Zhang, Yongfu Yu, Yuan Ma, Yihua Sun, Jiaqing Xiang, Yike Li, Haiquan Chen

**Affiliations:** 1 Departments of Thoracic Surgery and State Key Laboratory of Genetic Engineering, Fudan University Shanghai Cancer Center, 270 Dong’an Road, Shanghai, 200032 China; 2 Institute of Thoracic Oncology, Fudan University, Shanghai, China; 3 Department of Oncology, Shanghai Medical College, Fudan University, Shanghai, China; 4 Department of Pulmonary Medicine, Shanghai Xuhui Central Hospital, Zhongshan-Xuhui Hospital, Fudan University, Shanghai, 200031 China; 5 Department of Biostatistics, School of Public Health, and The Key Laboratory of Public Health Safety of Ministry of Education, Fudan University, Shanghai, China; 6 Chinese Institute for Brain Research, Beijing, China; 7 Department of Otolaryngology-Head and Neck Surgery, Vanderbilt University Medical Center, 1211 Medical Center Dr, Nashville, TN 37232 USA

**Keywords:** Machine learning, Esophageal squamous cell carcinoma, Recurrent laryngeal nerve, Lymph node metastasis

## Abstract

**Purpose:**

Esophageal squamous cell carcinoma (ESCC) metastasizes in an unpredictable fashion to adjacent lymph nodes, including those along the recurrent laryngeal nerves (RLNs). This study is to apply machine learning (ML) for prediction of RLN node metastasis in ESCC.

**Methods:**

The dataset contained 3352 surgically treated ESCC patients whose RLN lymph nodes were removed and pathologically evaluated. Using their baseline and pathological features, ML models were established to predict RLN node metastasis on each side with or without the node status of the contralateral side. Models were trained to achieve at least 90% negative predictive value (NPV) in fivefold cross-validation. The importance of each feature was measured by the permutation score.

**Results:**

Tumor metastases were found in 17.0% RLN lymph nodes on the right and 10.8% on the left. In both tasks, the performance of each model was comparable, with a mean area under the curve ranging from 0.731 to 0.739 (without contralateral RLN node status) and from 0.744 to 0.748 (with contralateral status). All models showed approximately 90% NPV scores, suggesting proper generalizability. The pathology status of chest paraesophgeal nodes and tumor depth had the highest impacts on the risk of RLN node metastasis in both models.

**Conclusion:**

This study demonstrated the feasibility of ML in predicting RLN node metastasis in ESCC. These models may potentially be used intraoperatively to spare RLN node dissection in low-risk patients, thereby minimizing adverse events associated with RLN injuries.

## Introduction

Esophageal carcinoma is the sixth most common cancer worldwide, resulting in an estimated total of 450,000 deaths per year [1]. Esophageal squamous cell carcinoma (ESCC) is a major histologic subtype that is most prevalent in East Asian and Middle Eastern regions. The lymph nodes along the recurrent laryngeal nerves (RLNs), located bilaterally in the tracheoesophageal grooves, have been shown as one of the most common sites of tumor metastasis in thoracic ESCC [2–9]. The reported incidence rate ranged from 20 to 40%, depending on the location and the stage of the tumor [3].

The current standard of care treatment to thoracic ESCC is surgery, which requires removal of the esophagus, reconstruction of the upper digest tract, and dissection of the upper mediastinal lymph nodes including those along bilateral RLNs [3, 10]. Iatrogenic injury to the RLN commonly occurs during this invasive procedure, with the incidence of as high as 69% [11]. RLN injury leads to vocal fold paresis or paralysis, causing hoarseness, stridor, aspiration pneumonia, or dyspnea in these patients. In particular, patients with bilateral vocal fold paralysis can suffer from severe dyspnea that may require long-term tracheostomy. These complications may significantly impair patients’ quality of life and even result in deaths [3].

As more than half of operable ESCC patients in fact have no RLN lymph node metastasis, they would benefit from selective dissection sparing these lymph nodes. It has been shown that enhanced computed tomography (CT) can reliably predict positive tumor metastasis in RLN lymph nodes that are greater than 6 mm in the short axis diameters [4, 12, 13]. However, imaging becomes much less effective in detecting occult metastasis in those smaller-size nodes. Positron emission tomography/CT only exhibited a low sensitivity of 45% in a recent prospective study of ESCC patients [14]. Ultrasound guided fine needle biopsy is not routinely applied to the RLN lymph nodes due to the technical challenges and the invasive nature of this procedure [15, 16]. As a result, the current treatment consensus for resectable ESCC suggest a systemic lymph node dissection including bilateral RLN nodes in all patients with the goal to minimize tumor recurrence [3, 17–21], as recurrence leads to extremely poor prognosis [7, 22–24]. In this regard, an effective prediction model for RLN lymph node metastasis is expected to promote personalized treatment decision-making by preventing unnecessary iatrogenic RLN injuries without increasing the risk of tumor recurrence. It can also guide the selection of dissection approaches if the risk of RLN node metastasis on each side can be assessed.

Machine learning (ML) is a subset of artificial intelligence that enables computers to learn from historical data and make predictions about new data using the information learned. With the advent of the big data era, ML has been increasingly applied to perform predictive modeling in medicine [25]. It has been shown to yield equivalent or superior outcomes compared to human judgment and traditional strategies in various tasks, such as disease detection, diagnosis, and prognosis prediction [26]. ML makes minimal assumptions about the characteristics of data, and therefore is effective even when the data are obtained without a controlled arm or in the presence of complicated nonlinear interactions among predictor variables [27]. Yet, ML has not been applied to predict RLN node metastasis in thoracic ESCC. The primary obstacle is the lack of a large-sized dataset with pathology-confirmed lymph node status.

This study was to investigate ML in prediction of RLN lymph node metastasis in patients with thoracic ESCC. To achieve this goal, a large-size, monocentric dataset was retrospectively collected and used to train and validate ML algorithms. Results obtained from this study should not only suggest the feasibility of ML for this task, but also provide insights into the clinical value of these models in personalized surgical planning of ESCC.

## Patients and methods

### Data collection

This study was conducted in full accordance with Good Clinical Practice and Declaration of Helsinki. Ethical approval and informed consent were waived by the Institutional Review Board of Fudan University Shanghai Cancer Center due to the retrospective study design. A medical record search was performed to identify patients with ESCC who were evaluated and surgically treated at this institute from January 2006 to December 2018. Detailed information including preoperative workups, indications and contraindications of surgery, and surgical approaches has been described in previous publications [7, 9, 14, 28, 29].

Patients from this cohort were eligible for the current study if they: (1) underwent a complete resection of thoracic esophageal cancer and a systemic lymphadenectomy along the esophagus, including dissection of the RLN lymph nodes on at least one side; (2) had a pathology-confirmed diagnosis of ESCC and the pathology report of the resected lymph nodes. Patients were excluded if: (1) they had received any preoperative treatment, such as chemotherapy and/or radiotherapy; (2) the short axis diameter of RLN lymph node on either side was measured greater than 6 mm on contrast-enhanced CT, as these patients all received neoadjuvant therapies [4, 12, 13]; or (3) there were any missing data.

The variables of interest included patients’ baseline characteristics (sex, age, body mass index), clinical information (history of smoking, alcohol use, family history of cancer, family history of esophageal cancer), and tumor’s histopathologic features (tumor location, grade, size, invasion depth, and the presence of any positive paraesophageal node in the chest [excluding RLN nodes] and the abdomen, respectively). These variables represented patient characteristics that were generally available and potentially associated with the outcome of interest in this study. In addition, these pieces of information could reasonably be obtained before the decision of RLN lymph node dissection was made. The outcome variable was the presence or absence of metastasis in the RLN lymph node on the target side. All data were deidentified before analysis.

### Data preprocessing

All categorical variables were encoded using the following standard protocol. Ordinal variables were converted to integer values from 1 through k, and nominal variables were encoded using a one-hot approach. A fivefold cross-validation method was applied to train and test the ML algorithms. Specifically, the dataset was split into an 80% training set and a 20% test set in a random, stratified fashion. This process was repeated for 5 times, each of which resulted in a completely distinct test set. During training, a random selection of 20% data from the training set were used to validate the models.

### Model development

The first task was designed to predict the risk of RLN lymph node metastasis on each side. A ground truth label of either metastasis- positive (1) or negative (0) was assigned to each RLN lymph node based on the pathology report. A total of 14 predictor variables were used, including all the patient- and tumor- related features, in addition to the target side. Five ML algorithms, including logistic regression, support vector machine (SVM), random forest (RF), extreme gradient boosting (XGBoost), and light gradient boosting machine (LightGBM), were trained in a binary classification task [30–32]. Grid search was first performed to obtain the optimal hyperparameters for each algorithm. In each cross-validation fold, each algorithm was trained to achieve convergence. The cutoff threshold of each model was determined at a level that yielded at least 90% negative predictive value (NPV). NPV is the ratio of true negative to the sum of true negative and false negative. This criterion was set to emphasize the model’s ability in correctly ruling patients out of RLN lymph node dissection. In other words, a patient who was predicted negative by the model should be at least 90% truly metastasis-free in the RLN nodes on the target side. The threshold was obtained on the validation set and applied to the test set.

The second task was extended from the first to predict the risk of RLN lymph node metastasis on the contralateral side, on condition that the metastatic status on the ipsilateral side was available. This task mimicked a clinical situation where the RLN lymph node on one side has been dissected and the frozen-section pathology is obtained, a decision should be made intraoperatively whether to continue dissection on the opposite side. For the second task, the predictor variables included the pathological status of the RLN lymph node on one side as well as all the features used in the first model. Only a subset of patients with pathology results of bilateral RLN lymph nodes were eligible. The rest of methods, including assignment of ground truth labels, types of ML algorithms, hyperparameter tuning and determination of thresholds, remained consistent as in the first task.

### Model testing

The classification performance of each model was evaluated on the test data by a set of metrics including accuracy, sensitivity, specificity, NPV, and the area under the receiver operating characteristic (AUROC). In particular, these scores were measured at the threshold predetermined during the training process.

### Feature importance

The importance of each predictor variable in a model was assessed by the permutation score on the test set. This score is defined as the decrease in model performance when all values of a given variable are randomly shuffled. Specifically, this procedure breaks the relationship between the feature and the outcome, therefore the magnitude of model performance drop is indicative of how much the model depends on this particular feature. For each variable in a model, this process was repeated for 100 times to obtain an average score.

### Statistics

Descriptive statistics were applied to characterize the baseline features of this dataset. The receiver operating characteristic curve of a model was created by plotting the true positive rate against the false positive rate at different thresholds. The AUROC score was measured by the entire area underneath the curve. The numbers of cases correctly and incorrectly classified by the framework were displayed in confusion matrices. Accuracy was measured by (true positive + true negative) / total sample size; sensitivity by true positive / (true positive + false negative); specificity by true negative / (true negative + false positive); and NPV by true negative / (true negative + false negative). Results were averaged over 5 cross-validation folds and expressed as mean ± standard deviation. All statistical analyses were performed using Python (Python Core Team, 2021) and Excel (Microsoft Corporation, Redmond, WA).

## Results

### Baseline characteristics

The dataset contained a total of 3352 East Asian patients that met the inclusion criteria for this study. The patient population consisted of 78.9% male and had an average age of 61.2 ± 7.77 years (mean ± standard deviation). Over 60% patients reported history of smoking (64.3%) and/or alcohol use (61.4%). Family history of cancer was documented in approximately a quarter of patients. In this cohort, the majority of tumors were moderately differentiated (61.5%) and located in the middle portion of the esophagus (64.9%). Over two thirds of tumors had invaded the muscle or the outer layer. There was a total of 38,552 lymph nodes harvested and pathologically assessed, averaging 11.5 per patient. Metastases were found in 12.1% lymph nodes (*n* = 4663) and 45.3% patients (*n* = 1519). Dissection of RLN lymph nodes was performed in 99.7% patients on the right side, and 96.9% on the left. Seventeen percent of the right RLN lymph nodes and 10.8% of the left were positive (Table 1).

**Table 1:** Baseline characteristics of the dataset

Characteristics n (%)	Patient count or mean	% or SD
	(*n* = 3352)	
Gender, n (%)		
Female	708	21.1%
Male	2644	78.9%
Age (mean, years)	61.24	7.77
BMI (mean, kg/m^2^)	22.86	2.71
Smokers, n (%)	2156	64.3%
Alcohol, n (%)	2057	61.4%
Family history of cancer, n (%)	816	24.3%
Family history of ESCC, n (%)	531	15.8%
Tumor location, n (%)		
upper	319	9.5%
middle	2176	64.9%
lower	857	25.6%
Pathologic T stage, n (%)		
Tis	44	1.3%
T1a	201	6.0%
T1b	801	23.9%
T2	1192	35.6%
T3	1103	32.9%
T4a	11	0.3%
Tumor grade, n (%)		
Gx	51	1.5%
G1	292	8.7%
G2	2062	61.5%
G3	947	28.3%
Tumor size (mean ± SD, cm)	3	1.5
Pathologic N stage, n (%)		
N0	1833	54.7%
N1	906	27.0%
N2	441	13.2%
N3	172	5.1%
Thoracic LN, n (%)		
Positive	748	22.3%
Negative	2604	77.7%
Abdomen LN, n (%)		
Positive	755	22.5%
Negative	2597	77.5%
Pathology-confirmed RLN LN, n (%)		
Left	3249	
Positive	351	10.8%
Negative	2898	89.2%
Right	3344	
Positive	569	17.0%
Negative	2775	83.0%

*SD* standard deviation, *BMI* body mass index, *ESCC* esophageal squamous cell carcinoma, *LN* lymph node, *RLN* recurrent laryngeal nerve

### Model performance

#### Task 1

The best hyperparameters for each algorithm is shown in Table 2. On average, the performance of all five ML models was comparable in every metric (Table 3). The mean AUROC score ranged from 0.731 (SVM) to 0.739 (RF), with only a 0.008 difference at most. All models showed NPVs that were consistent with the 90% criterion, suggesting a proper generalizability from the training data to the test data.

**Table 2:** Best hyperparameters settings for each algorithm

Models		Hyperparameters
	Logistic regression	‘C’: 0.1, ‘solver’: ‘saga’
Task 1	RF	max_depth’: 15, ‘max_features’: 3, ‘min_samples_leaf’: 10, ‘min_samples_split’: 2, ‘n_estimators’: 800
	SVM	‘C’: 0.01, ‘gamma’: ‘scale’, ‘kernel’: ‘linear’
	XGBoost	‘colsample_bytree’: 0.3, ‘eta’: 0.1, ‘gamma’: 0.0, ‘max_depth’: 7, ‘min_child_weight’: 7
	LightGBM	‘colsample_bytree’: 0.4, ‘gamma’: 0.0, ‘learning_rate’: 0.1, ‘min_child_samples’: 20, ‘num_leaves’: 5
Task 2	Logistic regression	‘C’: 0.1, ‘solver’: ‘liblinear’
	RF	‘max_depth’: 20, ‘max_features’: 10, ‘min_samples_leaf’: 1, ‘min_samples_split’: 2, ‘n_estimators’: 600
	SVM	‘C’: 0.01, ‘gamma’: ‘scale’, ‘kernel’: ‘linear’
	XGBoost	‘colsample_bytree’: 0.4, ‘eta’: 0.1, ‘gamma’: 0.2, ‘max_depth’: 3, ‘min_child_weight’: 5
	LightGBM	‘colsample_bytree’: 0.5, ‘gamma’: 0.0, ‘learning_rate’: 0.1, ‘min_child_samples’: 5, ‘num_leaves’: 50

These model-specific hyperparameters represent the global settings optimized for controlling the learning process of each model [30–32]

*RF* random forest, *SVM* support vector machine, *XGBoost* extreme gradient boosting, *LightGBM* light gradient boosting machine

**Table 3:** Performance metrics of each model obtained from five-fold cross-validation and expressed as mean ± standard deviation

Models		Accuracy	Sensitivity	Specificity	NPV	AUROC
	Logistic regression	80.5 ± 1.9%	36.1 ± 6.9%	87.7 ± 3.2%	89.5 ± 0.7%	0.735 ± 0.005
	RF	80.6 ± 2.4%	38.0 ± 4.6%	87.5 ± 3.5%	89.7 ± 0.4%	0.739 ± 0.010
Task 1	SVM	79.5 ± 2.5%	38.4 ± 8.4%	86.1 ± 4.2%	89.6 ± 0.7%	0.731 ± 0.008
	XGBoost	80.9 ± 1.7%	37.7 ± 2.9%	87.9 ± 2.3%	89.7 ± 0.3%	0.732 ± 0.011
	LightGBM	81.1 ± 1.0%	34.9 ± 2.6%	88.6 ± 1.3%	89.4 ± 0.3%	0.737 ± 0.012
	Logistic regression	80.4 ± 2.2%	40.0 ± 7.2%	86.8 ± 3.7%	90.1 ± 0.7%	0.748 ± 0.017
	RF	80.8 ± 3.7%	43.7 ± 7.5%	86.7 ± 5.2%	90.6 ± 0.8%	0.744 ± 0.019
Task 2	SVM	80.6 ± 1.7%	39.5 ± 6.0%	87.1 ± 2.8%	90.1 ± 0.6%	0.746 ± 0.017
	XGBoost	80.7 ± 2.8%	39.3 ± 7.0%	87.3 ± 4.3%	90.1 ± 0.6%	0.746 ± 0.018
	LightGBM	80.5 ± 2.0%	44.6 ± 7.9%	86.2 ± 3.5%	90.7 ± 0.9%	0.748 ± 0.017

*RF* random forest, *SVM* support vector machine, *XGBoost* extreme gradient boosting, *LightGBM* light gradient boosting machine, *NPV* negative predictive value, *AUROC* area under the receiver operating characteristic

**Fig. 1:**
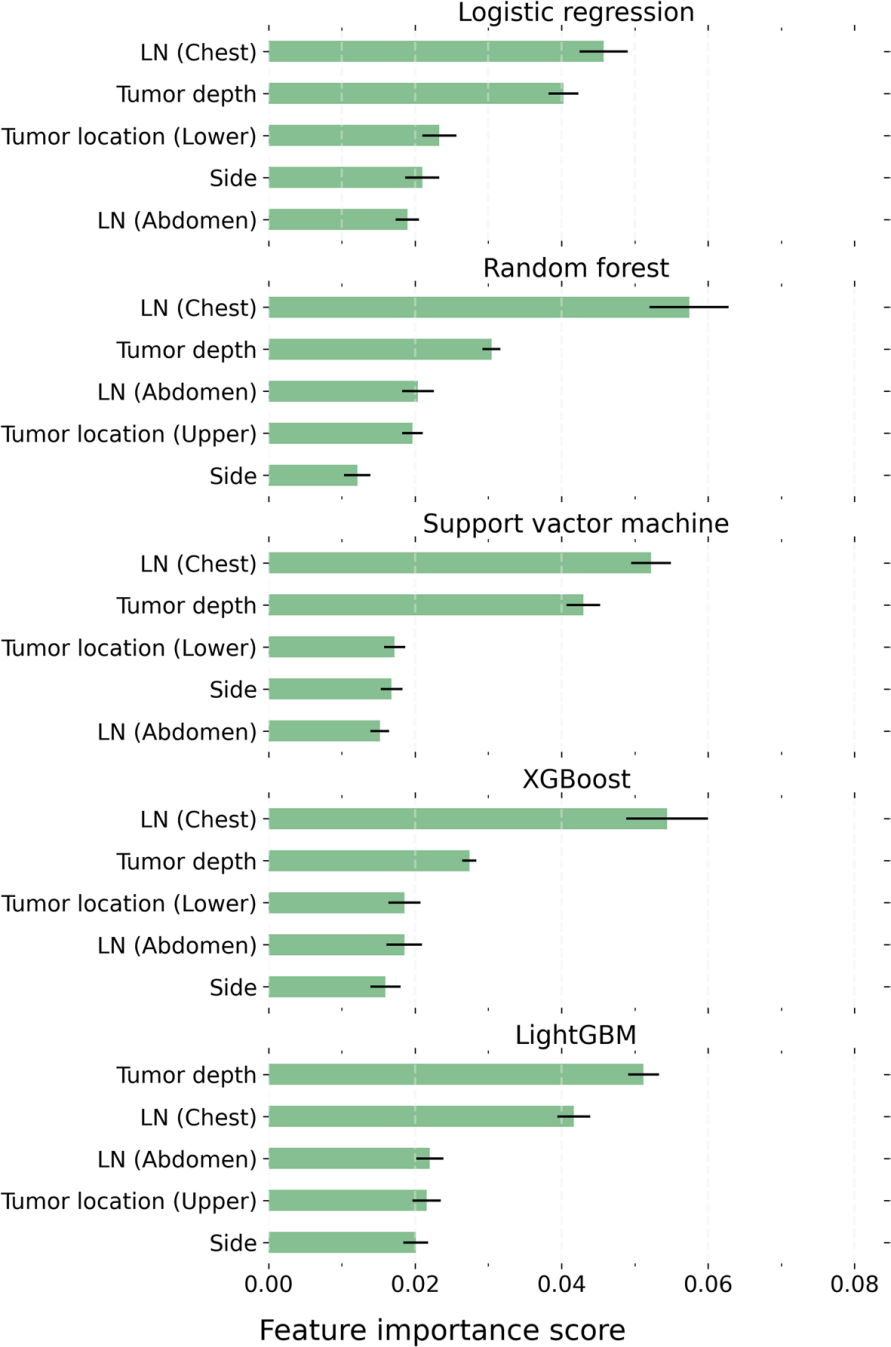
Critical features for task 1 models. The error bars indicate the standard error of the mean. LN: lymph node

The top 5 critical features for RLN node metastases were consistent across all models (Fig. 1), including the pathology status of other paraesophageal lymph nodes in the chest, tumor invasion depth, tumor location, target side, and the pathology status of abdominal lymph nodes. In particular, the pathology status of other paraesophageal lymph nodes in the chest ranked first in 4 of the 5 models, and second in the other.

#### Task 2

Similarly, all five models demonstrated almost equivalent performance in predicting RLN nodal metastasis on the contralateral side. The mean AUROC score was between 0.744 and 0.748, suggesting an average 0.005 to 0.015 improvement in model predictability with the additional feature of ipsilateral RLN lymph node status. The NPV values were all consistent with the 90% goal.

**Fig. 2:**
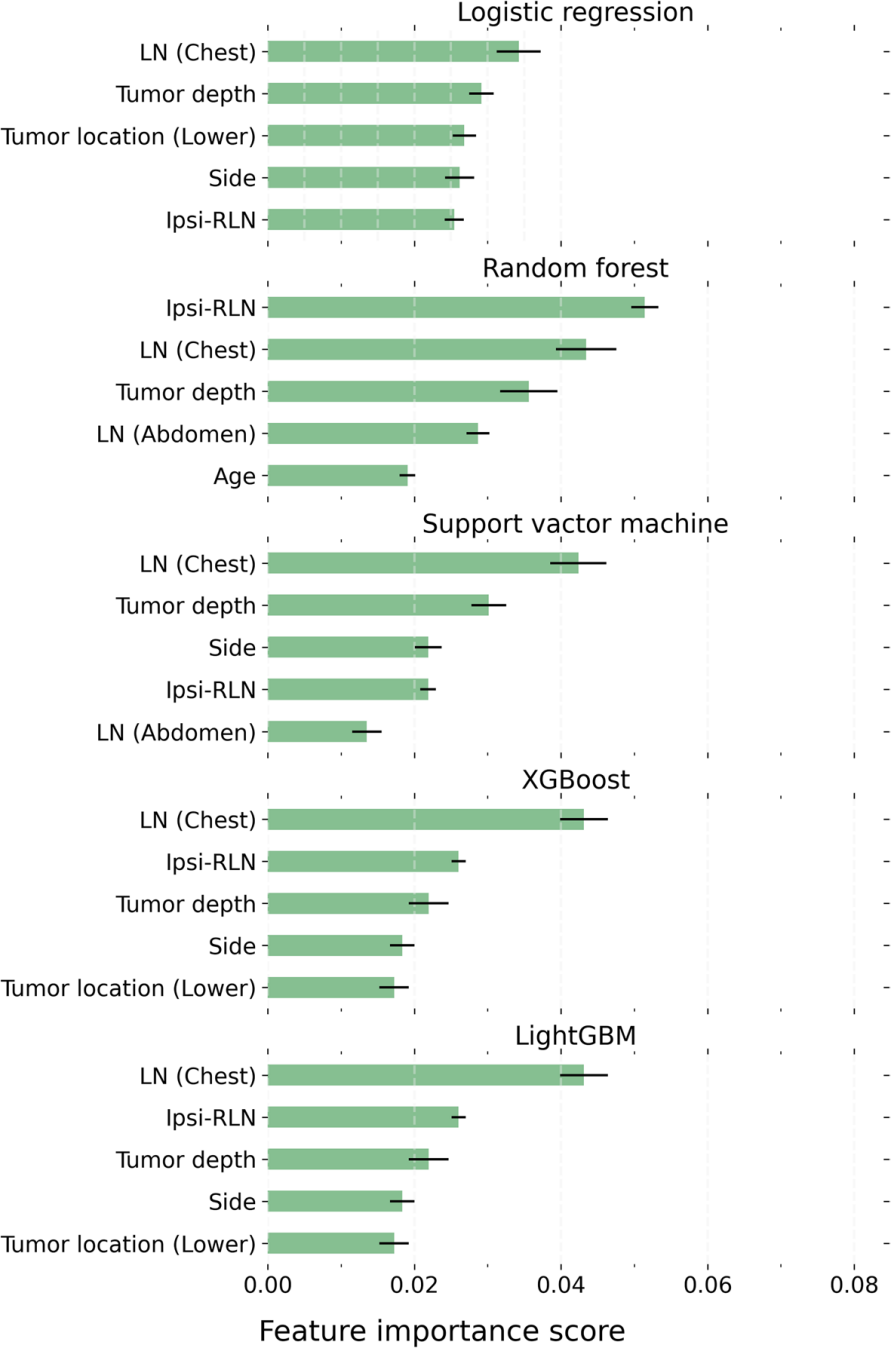
Critical features for task 2 models. The error bars indicate the standard error of the mean. LN: lymph node; Ipsi-RLN: Ipsilateral side of the recurrent laryngeal nerve lymph node

The top 5 critical features for contralateral RLN node metastases are displayed in Fig. 2. Three features were present in all models, including the pathology status of other chest lymph nodes, tumor invasion depth, and the ipsilateral RLN lymph node status. Other critical variables that were present in some models included tumor location (3), abdominal lymph node status (2), target side (4), and age (1).

## Discussion

Assessing the risk of nodal metastasis is beneficial for guiding surgical planning in patients with operable ESCCs. This study demonstrated the feasibility of ML in predicting tumor metastases in bilateral RLN lymph nodes. Models were developed to predict the risk of the target and the opposite side, respectively, based on patients’ baseline features and pathological findings. Each model showed adequate predictability with approximately 90% NPV that is practically meaningful. They can be sequentially implemented into a clinical workflow for intraoperative decision-making. To our best knowledge, this is the first ML study that investigates metastases of thoracic ESCCs in the RLN lymph nodes. This dataset also represents by far the largest monocentric cohort of patients that receive only surgical interventions and contain pathology results of the dissected lymph nodes. A fivefold cross-validation approach and a comprehensive set of performance metrics allow unbiased evaluation of the ML models. Assessment of the feature importance also yields informative findings for future research works. These advantages altogether suggest the validity of the outcomes and the feasibility of ML for such tasks. This study also lends support to the possibility of ML in guiding the prevention of important adverse events. With the potential of generating timely and reliable risk predictions, the role of ML in clinical care should grow rapidly in this big data era. In the future, ML is expected to become an integral part of routine clinical practice, and to notably promote personalized medicine.

Metastases of the thoracic ESCCs are frequently seen in lymph nodes of the neck, chest and upper abdomen. Specifically, tumor cells metastasize to distant nodes from the primary lesion through the rich longitudinal lymphatic vessel plexus in a “skip” and unpredictable fashion [3]. Previous efforts have been made to develop non-invasive methods for evaluating lymph node metastasis in ESCCs. By far, the lymph node size measured in the short axis from the enhanced high-resolution CT scan has been demonstrated as an easy and adequate indicator for metastases in the RLN nodes. In a recent study with 307 ESCC patients, a cut-off threshold of 6.5 mm yielded 50% sensitivity and 83.4% specificity in the right RLN nodes [33]. Another study based on a 5.5 mm threshold reported 64% sensitivity and 75% specificity in the left RLN nodes of 94 patients [13]. However, the high specificity and low sensitivity scores suggest this approach may only be desirable to rule in patients for removal of the RLN nodes. As 36 – 50% (i.e. 1 – sensitivity) of the metastases are present in the RLN nodes that measure less than the cut-off size and therefore missed by this approach, it is not reliable to rule patients out of lymph node dissection. In fact, these occult metastases in RLN nodes with normal or near normal radiologic appearances cannot be effectively detected by any imaging techniques neither. Recent studies of ESCC patients demonstrated low accuracies in nodal staging by either PET/CT scan [14] or endoscopic ultrasound [15]. Due to the lacking of any reliable approach for predicting occult metastasis and the extremely poor prognosis of recurrent ESCC, the current clinical guideline suggests an extensive lymphadenectomy for all operable patients even though it could result in additional trauma and complications. In this regard, a model that can reliably rule negative patients out of RLN node removal is clinically beneficial to account for the limitations of the radiologic approach and to minimize the dissection-associated complications. To the best of our knowledge, this is the first study on evaluating occult metastasis of ESCC in the RLN lymph nodes. The models developed in this study fill the aforementioned gap and may potentially supplement the existing methods in a two-stage clinical decision-making process. Specifically, the approach based on radiologic measurement may be employed preoperatively to determine patients who should require removal of the RLN nodes, and the current ML models further guides any intraoperative refinement in lymphadenectomy based on the frozen pathology results from other dissected nodes. With this two-model strategy and the 90% NPV scores of the second model, it is expected a high percentage of node-negative patients should be correctly identified and benefit from the selective dissection that spares the RLN nodes.

In this study, it was revealed by multiple models that some pathological features were universally critical to the risk of nodal metastasis. The presence of other positive nodes in the chest was one of the primary risk factors for a positive RLN node in both tasks. This finding can be supported by the current knowledge on esophageal anatomy. Specifically, once the tumors spread to any other thoracic lymph nodes, the likelihood of the RLN nodes being involved also increases as the superficial lymphatic vessels of the proximal esophagus have abundant direct connections with the RLN nodes [34]. Tumor location and the invasion depth appeared to be the other two important risks factors, which were also consistent with previous findings [10, 35, 36]. Further, it was revealed in this study that the status of the ipsilateral RLN node contributed to only a minor improvement in model predictability for the outcome of the contralateral side. This finding, in addition to the low importance scores of the side feature in these models, suggests tumor metastasis in ESCC is largely side-independent. It is supported by previous studies showing the lymphatic drainage of the esophagus is longitudinal with no evidence of direct anatomical connection between the left and the right RLN lymph nodes [34, 37]. It is noteworthy that, the feature importance scores are model-dependent and subject to the data characteristics. Therefore, these findings are only interpreted qualitatively. Still, they should be indicative of developing hypotheses and designing future research works to better understand the pattern of lymph node metastasis in ESCC.

ML has been increasingly applied in medicine as a powerful tool for data-driven research [25]. Compared to the traditional statistics, it excels at capturing the non-linear and complicated interactions among a large number of variables [27]. Many ML algorithms, including the classic RF, SVM and k-nearest neighbors, as well as the state-of-the-art models like XGBoost and LightGBM, have been explored in a variety of studies [38–41]. The performances of these models vary by the task and the data. Model comparison is necessary to obtain the optimal strategy for a specific task or on a particular dataset. In the current study, model predictability assessed by the AUROC score was comparable in both tasks for all ML models, suggesting each algorithm should be equivalent and sufficient for prediction of RLN node metastasis. In particular, the logistic regression, a classic statistical approach, demonstrated non-inferior performance compared to other ML algorithms. This finding is consistent with a few previous studies that showed ML algorithms yielded only marginal or even no performance gain over the standard regression models [38–42]. The most likely explanation is the sample size and/or the number of features were relatively small in these studies to achieve the optimal performance by ML, as most ML algorithms are data hungry (e.g. millions of data). Meanwhile, there is another concern that a small number of the events of interest may limit the potentials of ML models in discovering the underlying patterns from these rare “positive” cases. This situation is in fact common in many diseases with low incidence or prevalence rates [38, 43]. For example, the positive RLN nodes in this study only accounts for less than 20% on each side, which could result in a model biased towards a negative prediction. It is therefore implied that traditional regression models should continue to play a key role in disease risk prediction, especially when a small sample size, limited predictor variables, or a highly imbalanced dataset is encountered. The fact that some of these ML models are subject to the risk of overfitting and the lack of interpretability further favors the use of simple regression models, which can be translated to explainable equations.

Some limitations should be noted in this study. First, only a monocentric dataset was obtained. Although this dataset represents the largest ESCC cohort with natural metastatic progression that is unaltered by any induction therapies, it may still be subject to certain biases, such as race- and region-related factors. Therefore, the generalizability of these models may require further validation on external datasets. Second, only a small number of variables were employed for prediction. Although each feature was reasonably selected by availability and the potential association with the outcome, additional variables may still be necessary to reach a more reliable prediction. Specifically, a comprehensive model may yield better performance by mimicking clinician’s decision-making strategy based on all useful information including history, clinical presentations, lab results, imaging and pathology. Third, participants of this study underwent postoperative adjuvant therapy instead of preoperative neoadjuvant therapy. Although neoadjuvant therapy is more commonly used for advanced esophageal cancer in western countries, [1, 44] there are multiple studies suggesting that surgery plus adjuvant therapy leads to a similar 5-year overall survival rate for ESCC patients [2, 7, 45, 46] and even greater survival for those with metastatic lymph nodes [46, 47]. Therefore, adjuvant chemotherapy is the standard of care for ESCC patients with suspected lymph node metastases in most Chinese hospitals, as it can be guided by a more precise intraoperative staging and results in better patient adherence [48]. Nonetheless, the difference in treatment strategies may potentially limit the generalizability of these models. Future research works should be designed to address these limitations. For example, imaging findings can be added as predictor variables to improve the ML models; a deep learning framework may be developed for automatic radiology interpretations and applied in conjunction with these models; any predictive models should be evaluated on external data; and this technique can be applied to predict metastases in other lymph nodes or in other tumors. Progress is being made to collect more data and to test these models in a prospective study. The ultimate goal of this research line is to advance the understanding of lymph node metastasis in ESCC and to improve overall prognosis by minimizing unnecessary lymph node dissection.

## Conclusion

This study demonstrated the feasibility of ML in predicting RLN node metastasis in ESCC based on patients’ baseline and pathologic features. Logistic regression showed comparable performance to other ML models in both tasks. The presence of other positive nodes in the chest and tumor invasion depth were the top 2 most critical factors for prediction of RLN node metastasis. The resulting models may potentially be applied intraoperatively to guide the dissection of RLN lymph nodes. Future works should be conducted to improve these ML models by adding more predictor variables and to test them on external data.

## Data Availability

The datasets generated during and/or analyzed during the current study are available from the corresponding author on reasonable request.
